# Dermatofibrosarcoma Protuberans of the Breast in Man: An Extremely Rare Entity With a Review of the Literature

**DOI:** 10.1177/2324709619875634

**Published:** 2019-09-15

**Authors:** Malek Bouhani, Yasmine Fertani, Ines Zemni, Olfa Adouni, Amine Bouida, Riadh Chargui, Rahal Khaled

**Affiliations:** 1 Salah Azaiz Institute, Tunis, Tunisia

**Keywords:** dermatofibrosarcoma protuberans, breast, male

## Abstract

Dermatofibrosarcoma protuberans (DFSP) is a rare sarcoma of soft tissue representing about 1% of all tumors. In addition, DFSP occurs commonly on the trunk and extremities, and only a few cases of DFSP have been observed on the breast. In men, only 11 cases, including this case, have been reported. In this article, we present a case of left breast DFSP that occurred in a 44-year-old man. The physical examination revealed a left breast tender mass, which invaded the skin. The tumor was staged as T4b N0 M0. Mammography and sonography showed a suspect mass of the left breast. The biopsy and immunochemistry permitted the diagnosis of DFSP of the left breast. The patient had a left mammectomy, with free margins. He presents no evident sign of recurrence 7 months later.

## Introduction

Dermatofibrosarcoma protuberans (DFSP) was first described by Taylor in 1890 as a keloid sarcoma.^1^ Later, Ferrand identified it as a recurrent dermatofibroma.^2^ In 1924, Hoffman termed this entity as DFSP.^3^ DFSP is an uncommon soft tissue sarcoma that accounts for <5% of all soft tissue sarcomas during early or middle adult life.^4^ DFSP is a mesenchymal tumor that involves subcutaneous tissue and dermis.^4^ DFSP is predominant in the trunk and extremities.^5^ Breast involvement of DFSP is a rare situation and even rarer in men. In this article, we describe a case of DFSP that occurred in the male breast.

## Case Presentation

We report a case of a 44-year-old man, with a long history of smoking, who was referred to our department for a swelling on his left breast. His family history was unremarkable of breast cancer.

The anamnesis revealed the occurrence of left breast trauma, 3 years ago, and then he developed a breast soreness, which progressively increased in size.

**Figure 1.**
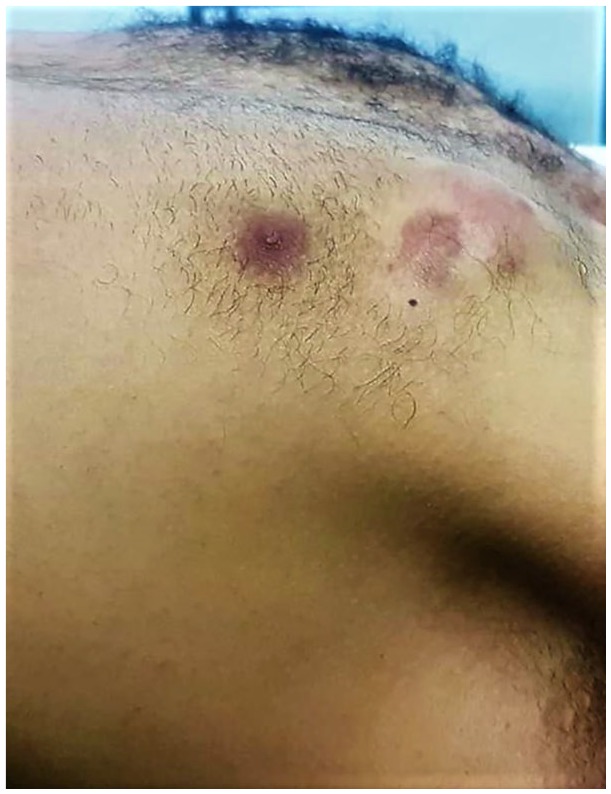
Clinical examination showing a firm, multinodular, irregular mass in the upper union quadrant of the left breast.

Clinical examination showed a firm, tender, mobile mass measuring 4 cm in the greatest diameter, located in upper union quadrant of the left breast and invaded the skin (Figure 1). No lymph node was palpable.

**Figure 2.**
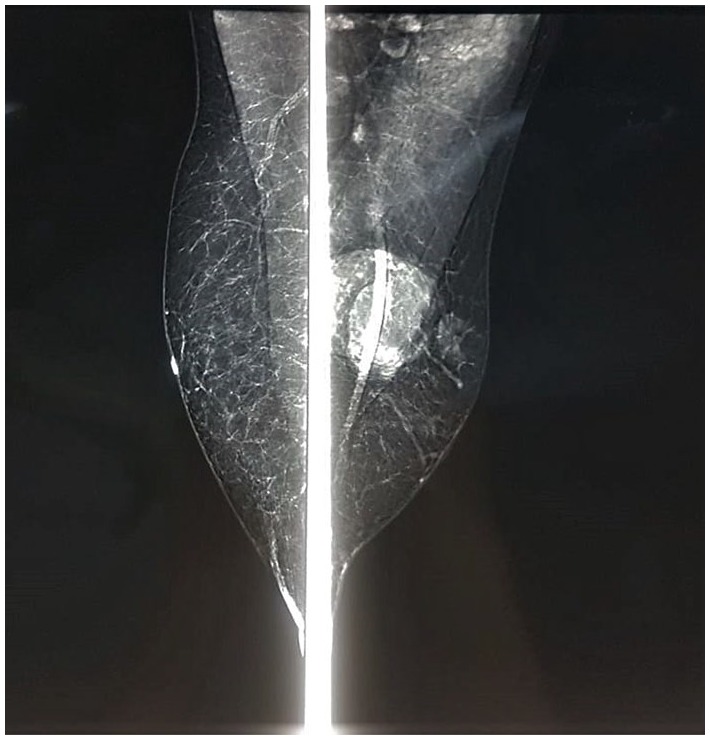
Mediolateral oblique mammography showed a subcutaneous oval mass with a smooth and sharp margin on the left breast, the right breast was normal.

Ultrasonography revealed an indistinct limited mass with central vascularization, measured 4 cm, and mammography demonstrated a nonhomogeneous and hyperdense lesion, which classified the tumor as a BI-RADS 4B (Breast Imaging Reporting and Data System 4B; Figure 2).

The patient had a core needle biopsy, and the histology concluded to a DFSP of the breast. Chest X-ray and ultrasound of the abdomen were performed, and there was no evidence of distant metastases. The tumor was ranked T4b N0 M0.

The patient underwent a left mammectomy with macroscopically free margins of 3 cm. The frozen section examination revealed a dendritic differentiation with an invasion of the pre-pectoral fascia despite macroscopic free limits. We did a re-excision removing localized pectoralis muscle fibers, and the frozen section examination showed free margins.

**Figure 3.**
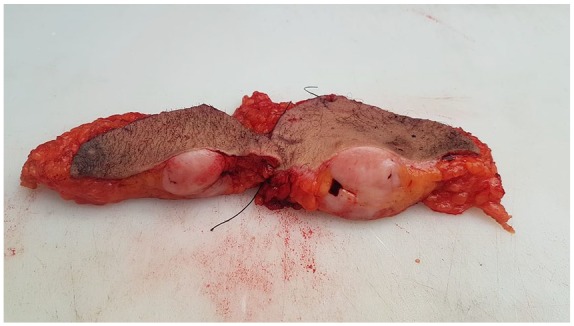
Whitish poorly limited nodular formation, measuring 4 cm in the largest dimension and bulging under the skin.

**Figure 4.**
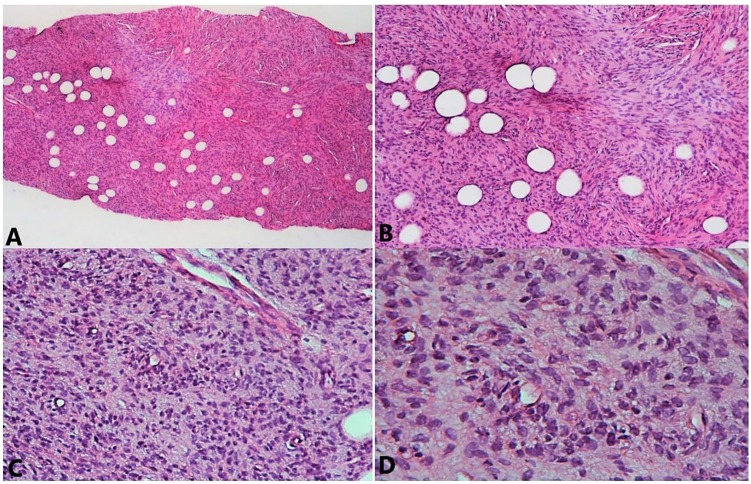
Progressive magnification in hematoxylin-eosin staining: (A) ×50, (B) ×100, (C) ×200, and (D) ×400. The tumor showed densely packed spindle cells arranged in focal storiform as well as herringbone pattern with myxoid areas in between and infiltration of tumor cells into subcutaneous fatty tissue, with rare figure of mitosis.

The histopathological examination revealed a whitish, indurated, poorly limited nodular formation, measuring 4 cm in the largest dimension and bulging under the skin (Figure 3). Histological examination showed mesenchymal proliferation made of short intertwined beams that recall in places the appearance of a honeycomb. The stroma was fibrous and sometimes myxoid (Figure 4).

Tumor cells were regular monomorphic with an elongated nucleus and poorly defined cytoplasm. Mitosis was rare. The tumor infiltrated the adipose tissue.

**Figure 5.**
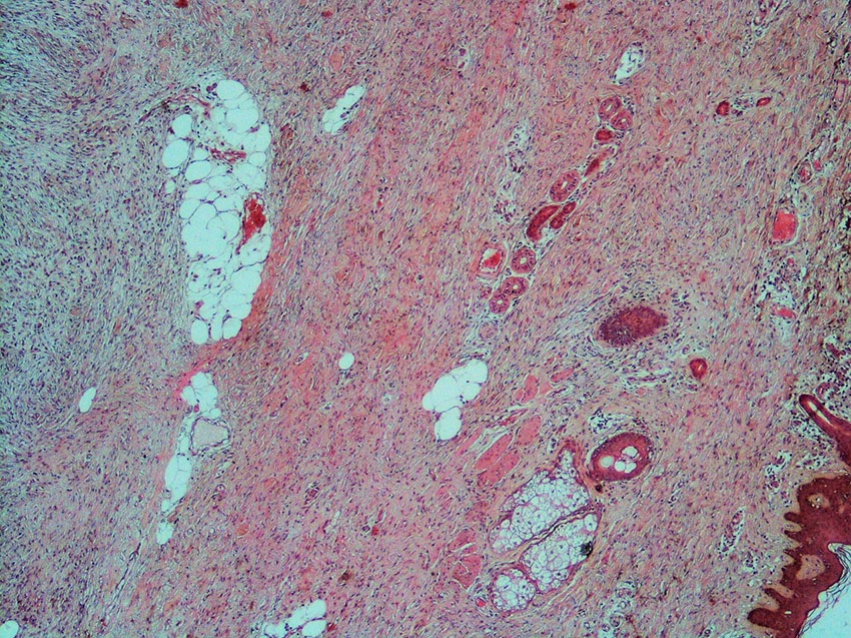
Tumor involving overlying skin.

This tumor extended from the papillary dermis to the muscular level without infiltrating it (Figure 5). The Ki 67% was estimated at 3%.

**Figure 6.**
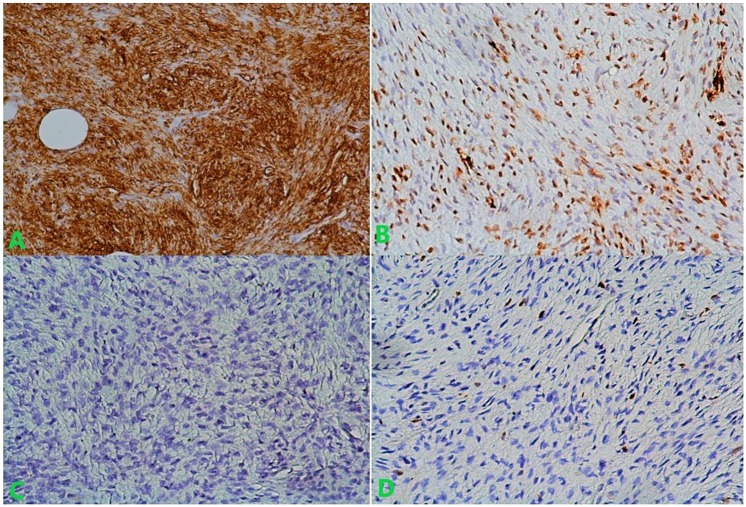
Magnification ×200, immunohistochemistry showed: (A) CD34 positivity in tumor cells, (B) focal positivity of SMA, (C) negativity of desmin, and (D) negativity of CK.

Immunochemistry showed a strong positivity to CD34, while smooth muscle actin was focally positive. CK and desmin were negative (Figure 6). Moreover, in the definitive histological examination, the surgical margins were 3 cm, and the shaved part of the muscle was free from tumor invasion, achieving a histologically deep free margin of 2 cm. The accurate diagnosis of DFSP of the left breast was established.

Post recovery of the patient was good. No locoregional recurrence or distant metastasis was found after 7 months of follow-up.

## Discussion

Dermatofibrosarcoma protuberans is a low-grade malignant soft tissue tumor that typically develops in the dermis and then extends to subcutaneous tissues and muscles.^6^ The median age of presentation is 38.5 years with a distribution approximately equal between males and females.^7^ The trunk and extremities are considered DFSP’s most frequent sites.^8^ However, its occurrence on the breast is very rare.^4^

We did an extensive review on PubMed and Google Scholar, and we founded 10 cases of DFSP of the breast in male patients from 1994 to 2019 (Table 1).^4,9-17^ To the best of our knowledge, this case represents the 11th case of the breast DFSP in men.

**Table 1.** Clinicopathological and Immunohistochemical Features of DFSP in the Male Breast in Isolated Case Reports.

Authors	Years	Age (Years)	Size (cm)	Side	IHC
Chen et al^9^	2009	41	4.5	Retroareaolar right breast	CD34+, vimentin+, Bcl-2−, S-100−, SMA−, desmin−, and EMA−
Park et al^10^	2011	36	—	Peri-areolar right breast	CD34+, factor XIIIa−
Akhtar et al^11^	2012	22	5	UQ, LOQ, and peri-areolar left breast	CD34+, SMA−, S-100−, and Bcl-2−
Prabhu et al^12^	2014	55	5	UOQ left breast	CD34+
Al Tarakji et al^4^	2015	27	4	LOQ right breast	CD34+, Bcl-2+, CD99+, β-catenin+, and vimentin+
Saikia et al^13^	2016	40	6.5	Central left breast	CD34+, SMA−, S100−, desmin−, CK−, and EMA−
Ezejiofor et al^14^	2017	13	12	UOQ right	CD34+, S-100−, pan cytokeratin−, and CD117−
Diwakar et al^15^	2018	35	5	LOQ right breast	CD34+, vimentin+, S-100−, and SMA−
Dhakal et al^16^	2018	48	13	Central left breast	CD34+
Murase et al^17^	2019	9	3	Peri-areolar left breast	CD34+
Current case	2019	44	4	UUQ left breast	CD34+, SMA focally +, CK−, and desmin−

Abbreviations: DFSP, dermatofibrosarcoma protuberans; IHC, immunohistochemistry; Bcl-2, B-cell leukemia-lymphoma; SMA, smooth muscle actin; EMA, epithelial membrane antigen; UQ, upper quadrant; LOQ, lower outer quadrant; UOQ, upper outer quadrant; UUQ, union upper quadrant.

The pathogenesis of DFSP remains unknown. DFSP was observed to occur in pre-traumatic areas, including vaccination sites, burn scars, tattoos, surgical scars, and radiotherapy.^15^ Trauma may initiate chronic inflammation that leads to immune system disorder and then malignant transformation of the dermis.^15^ This hypothesis is supported by genetic findings such as chromosomal translocation of t(17; 22) (q22; q13) between chromosomes 17 and 22, the fusion of gene COL6A3-PDGFD, and the fusion of gene COL1A1-PDGFB.^15,17^

Due to the rarity of this presentation, diagnosis could be misleading. The most common presentation in male’s breast is a mass with extensive nodules on the surface, most commonly about 1 to 5 cm in diameter.^4,9-17^ In fact, a breast mass could imitate a breast carcinoma, phyllode tumor, and myoepithelioma.^4,15^ In our case, the clinical appearance was nonspecific.

In mammography, DFSP appears as an irregular dense mass without fat or calcification.^18^ Ultrasound shows a heterogeneous or hypoechoic mass, irregular with hypervascularity of the affected areas in color Doppler. Magnetic resonance imaging can help determine the tumor’s infiltration depth.^15^ Due to the nonspecific imaging findings, fine needle aspiration is required.^4^

Histologically, monomorphic spindle cells arranged in a storiform pattern are distinctly composed. The intense staining for CD34 differentiates DFSP from myxoid liposarcoma.^4,9,10^ Our case showed diffuse strong positivity for CD34 and focal immunoreactivity for smooth muscle actin.

Surgery remains the treatment cornerstone. But surgeon has to keep in mind the oncologic outcomes and cosmetic issues in the male breast. Moh’s surgery provides maximum tissue conservation and better margins than wide local excision (WLE), but the local rate of recurrence is statistically comparable.^19^ Indeed, the lack of expertise in all centers leads to the use of the WLE technique with or without plastic reconstruction as the preferred surgery.^12^ The present case is unique due to the dendritic presentation and shows limits of the frozen section examination. Free macroscopic margins could not be enough even with large excision, as in our case, we found the invasion of the inferior limit despite free-looking anatomic barriers and underlying fascia.

Resection margins of at least 2 to 3 cm are recommended for treatment because the local recurrence rate is 20% to 50% in cases of incomplete resection.^4^

Adjuvant radiotherapy should be used to treat an unresectable lesion or resected lesion with a positive margin. Adjuvant radiotherapy may efficiently decrease the rate of local recurrence and prevent the mutilation and functional deficits resulting from repeated surgery.^20^

The use of imatinib as treatment for unresectable, recurrent, and/or metastatic DFSP for patients who are not eligible for surgery showed a response rate between 5.2% and 55.2%.^21^

Prognosis factors are increased age, a high mitotic index, and increased cellularity.^22^ Long-term follow-up is required owing to the high rate of recurrence.

Our case has been treated with a radical left mastectomy without any adjuvant treatment. Our literature search concludes to only 10 other cases of DFSP in the male breast. The mean age of these patients was 32.6 years with an extreme range from 9 to 55 years, and the mean tumor size was 7.1 cm with an extreme range from 3 to 13 cm. To the best of our knowledge, the present case is the 11th.

## Conclusion

The clinical presentation of DFSP in the male breast could imitate a breast carcinoma. Diagnosis requires biopsy with immunochemistry examination. WLE with 2 to 3 free margins reduces the rate of recurrence.
